# Cerebral Venous Sinus Thrombosis During Raloxifene Therapy: A Case Report

**DOI:** 10.7759/cureus.85486

**Published:** 2025-06-06

**Authors:** Shunsuke Kimura, Tadataka Mizoguchi, Yusuke Imamura, Naoki Tagawa, Kota Mori, Takahiro Kuwashiro, Hiroshi Sugimori

**Affiliations:** 1 Cerebrovascular Medicine and Neurology, National Hospital Organization Kyushu Medical Center, Fukuoka, JPN

**Keywords:** cerebral venous sinus thrombosis (cvst), intracranial hemorrhage (ich), osteoporosis, raloxifene, stroke, subcortical hemorrhage

## Abstract

A 79-year-old woman taking raloxifene for osteoporosis was admitted to our hospital with right-sided hemiplegia and seizures. Computed tomography (CT) of the head revealed a subcortical hemorrhage in the left frontal to parietal lobes. CT venography (CTV) showed a disruption of the superior sagittal sinus, leading to a diagnosis of cerebral venous sinus thrombosis (CVST). In the absence of other thrombogenic factors, raloxifene was considered a potential contributing factor. She received continuous intravenous heparin followed by warfarin, without clinical deterioration, and was later transferred to a rehabilitation hospital. Although venous thrombosis is a recognized adverse effect of raloxifene, CVST remains an uncommon manifestation. We therefore present this case to highlight the potential association.

## Introduction

Cerebral venous sinus thrombosis (CVST) is a rare cerebrovascular disorder, accounting for approximately 0.5% to 1% of all stroke causes [1]. The clinical presentation frequently includes headache and seizures due to elevated intracranial pressure, along with focal neurological deficits caused by venous infarction or cerebral hemorrhage. CVST predominantly affects younger women, with established risk factors including thrombophilia, oral contraceptive use, pregnancy, and the postpartum period [2,3]. Raloxifene, a selective estrogen receptor modulator (SERM) prescribed for postmenopausal osteoporosis, has been known to increase the risk of venous thrombosis, likely due to its estrogenic effects on hepatic coagulation pathways. These effects include significant reductions in plasma antithrombin activity, elevated plasma levels of coagulation factors VIII, XI, and XII, and decreased sensitivity to activated protein C in postmenopausal women [4-10]. However, to the best of our knowledge, no previous cases of CVST associated with raloxifene use have been reported. Herein, we present such a case.

## Case presentation

A 79-year-old woman with a history of dementia resided in a nursing facility. Her pre-onset modified Rankin Scale score was four, and she required a wheelchair for mobility due to her inability to walk independently. Her regular medications included raloxifene for osteoporosis, donepezil and memantine for Alzheimer’s disease, and quetiapine for behavioral and psychological symptoms of dementia. The exact duration of each medication was unknown. 

She developed right-sided weakness the day before and was kept under observation at her facility but was transferred to our hospital with right hemiplegia after experiencing a brief seizure the following day. On a neurologic examination, her level of consciousness was impaired; she opened her eyes in response to verbal stimulation but was unable to speak. No alterations in cranial nerve examination. No convulsions occurred, but oral automatisms were noted. The right upper and lower extremities showed no response to pain stimuli, although accurate assessment of paralysis and sensory deficits was difficult due to impaired consciousness. The National Institutes of Health Stroke Scale (NIHSS) score was 25. Laboratory tests showed an elevated D-dimer level, while complete blood counts, serum electrolytes, and glucose concentrations were within normal limits, with no evidence of dehydration (Table 1).

**Table 1:** Laboratory tests WBC: white blood cells; RBC: red blood cells; HGB: hemoglobin; HCT: hematocrit; MCV: mean corpuscular volume; MCH: mean corpuscular hemoglobin; MCHC: mean corpuscular hemoglobin concentration; PLT: platelet

Test/unit	Result	Normal range
WBC (/µL)	8.60 x 10^3^	3.3–8.6
RBC (/µL)	4.09 x 10^3^	4.35–5.55
HGB (g/dL)	12.4	13.7–16.8
HCT (%)	38.8	40.7–50.1
MCV (fL)	94.9	83.6–98.2
MCH (pg)	30.3	27.5–33.2
MCHC (g/dL)	32.0	31.7–35.3
PLT (/µL)	25.3 x 10^6^	158–348
Sodium (mmol/L)	141	138–145
Potassium (mmol/L)	4.2	3.6–4.8
Chloride (mmol/L)	105	101–108
Urea nitrogen (mg/dL)	11	8–20
Creatinine (mg/dL)	0.42	0.65–1.07
Glucose (mg/dL)	94	73–109
D-dimer (µg/mL)	6.2	<1
Antithrombin III (%)	81	80–130
Anticardiolipin antibodies (U/mL)	2.9	0–3.4
Lupus anticoagulant	1.0	<1.2
Homocysteine (nmol/mL)	10.7	5.3–15.2
Protein C (%)	137	64–146
Protein S (%)	105	56–126

**Figure 1:**
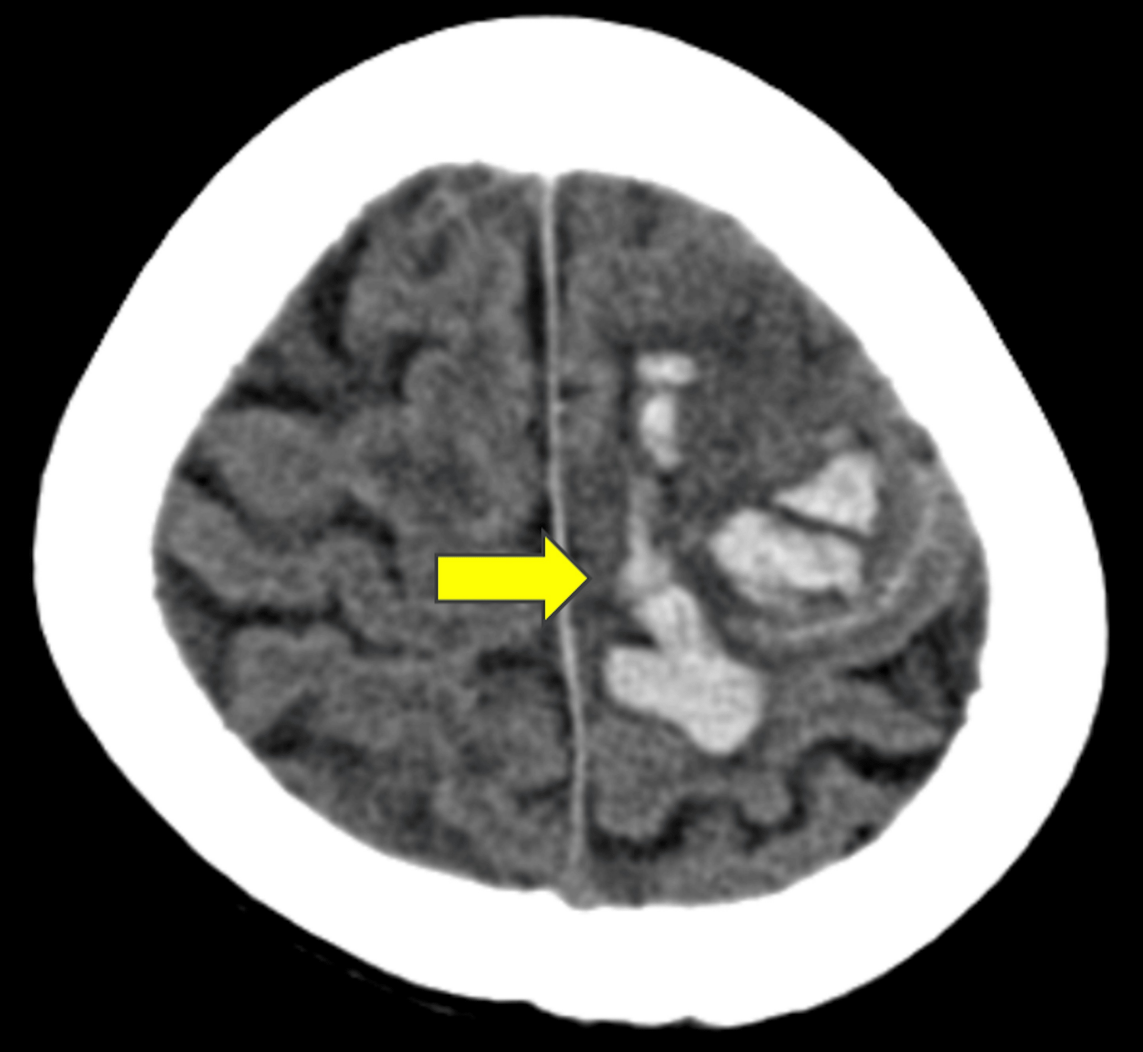
The patient's head CT on admission revealed subcortical hemorrhage in the left frontal to parietal lobes. CT: computed tomography

A computed tomography (CT) of the head revealed a subcortical hemorrhage in the left frontal to parietal lobes (Figure 1).

**Figure 2:**
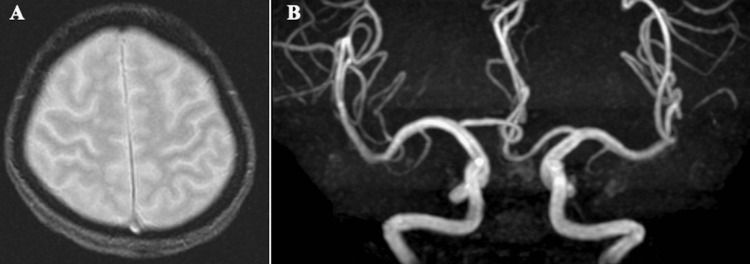
A. Magnetic resonance imaging of the head was performed 13 years prior to the patient's current presentation demonstrated no cerebral microbleeds on T2-weighted imaging; B. Magnetic resonance angiography of the anterior circulation showed no abnormal vessels or aneurysms.

A prior magnetic resonance imaging (MRI) performed 13 years earlier demonstrated no microbleeds on T2-weighted imaging, and magnetic resonance angiography (MRA) showed no vascular anomalies or aneurysms (Figure 2).

**Figure 3:**
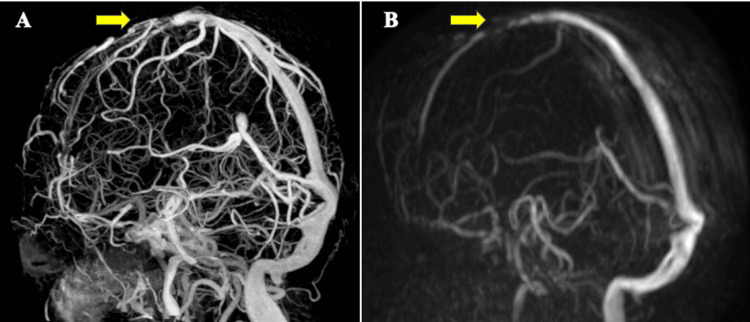
Computed tomography venography (CTV) of the head on admission and magnetic resonance venography performed 11 days later. A. CTV revealed a contrast defect in the superior sagittal sinus; B. Magnetic resonance venography performed 11 days later demonstrated slightly improved visualization of the superior sagittal sinus compared to the initial CTV.

Current head CT venography (CTV) revealed a contrast defect in the superior sagittal sinus (Figure 3A), confirming the diagnosis of CVST.

Head CT revealed no evidence of sinusitis or trauma, and lower extremity venous ultrasonography showed no signs of deep vein thrombosis. Whole-body CT showed no findings suggestive of malignancy, and there was no history of head trauma after admission to the facility. A comprehensive thrombophilia workup, including coagulation studies, antithrombin III, protein C, protein S, antiphospholipid antibodies (lupus anticoagulant and anticardiolipin antibodies), and homocysteine levels, was performed before treatment initiation and showed no abnormalities. Given the plausible association between raloxifene and CVST, the medication was immediately discontinued upon admission. Continuous intravenous infusion of unfractionated heparin was initiated, followed by oral warfarin starting on hospital day 4. The target prothrombin time (PT)-international normalized ratio (INR) range for warfarin therapy was set at 2.0-3.0, and the heparin infusion was discontinued on day 10 once this therapeutic range was achieved. Magnetic resonance venography (MRV) performed on day 11 showed partial recanalization of the superior sagittal sinus (Figure 3B). Although no clinical deterioration occurred after admission, the patient was left with severe neurological deficits and was transferred to another facility on day 17.

## Discussion

We report a case of CVST that occurred during raloxifene therapy. Raloxifene, a SERM commonly prescribed for osteoporosis in postmenopausal women, has been associated with an elevated risk of venous thromboembolism. To our knowledge, no previous reports have documented an association between raloxifene and CVST. Although a definitive causal relationship cannot be established, the temporal association and absence of other identifiable risk factors suggest a potential link. Unawareness of this potential association may result in delayed diagnosis or inappropriate continuation of raloxifene treatment even after the onset of CVST, thereby potentially worsening clinical outcomes. It is therefore essential for clinicians to recognize this rare but serious complication.

Raloxifene exerts tissue-selective estrogen-like effects on bone and serum lipids without apparent stimulatory effects on mammary or uterine tissues [4-8]. However, raloxifene therapy has been linked to an increased risk of venous thromboembolism [6,7], likely due to its estrogenic effects on hepatic coagulation pathways, including significant reductions in plasma antithrombin activity [9], elevated plasma levels of coagulation factors VIII, XI, and XII, and decreased sensitivity to activated protein C in postmenopausal women [10]. In contrast, raloxifene therapy has not been reported to increase the incidence of cardiovascular events or stroke, and no previous reports have identified a link between raloxifene and CVST [11]. Given that low-dose estrogen-containing oral contraceptive agents with similar estrogenic effects are well-established risk factors for CVST [2, 12], raloxifene may similarly increase the risk. Although CVST typically affects younger women, this case highlights its potential occurrence in postmenopausal women receiving raloxifene therapy.

As described above, although raloxifene has been associated with an elevated risk of venous thromboembolism, the limited number of documented CVST cases may be attributable to several factors. One potential explanation is underdiagnosis, because some patients may have developed CVST without clinicians recognizing it. While a previous study found no significant increase in ischemic stroke incidence with raloxifene compared to placebo, it did report a higher rate of fatal strokes; however, the mechanism underlying this increased fatality was not elucidated [11]. Unlike typical ischemic stroke or intracerebral hemorrhage, CVST can be fatal if not promptly diagnosed and treated. It is hypothetically possible that some fatal strokes among patients receiving raloxifene may have resulted from undetected CVST, although supporting evidence is currently lacking. Another explanation is that raloxifene may increase the risk of CVST, particularly in the presence of additional risk factors. In the present case, the patient’s severe dementia limited her mobility, likely resulting in prolonged bed rest and venous stasis, both of which may have contributed to CVST development. Although rare, a few cases of CVST have been reported in patients receiving tamoxifen, another SERM used in breast cancer treatment [13,14]. All these patients had malignancies, known risk factors for CVST, suggesting that SERM treatment may further increase CVST risk in predisposed individuals [2]. Additional studies are warranted to validate the observed association between raloxifene use and CVST and to clarify the contributing risk factors.

In this case, CVST was initially suspected based on elevated D-dimer levels, the absence of cerebral microbleeds on prior MRI (suggesting no evidence of cerebral amyloid angiopathy), and the lack of abnormal vessels or aneurysms on MRA. The diagnosis was ultimately confirmed by CTV. In cases of intracerebral hemorrhage (ICH) due to CVST, anticoagulation therapy is required in addition to standard antihypertensive treatment [15]. As anticoagulants are generally avoided in ICH, failure to identify underlying CVST can result in missed opportunities for appropriate treatment and lead to poor outcomes. Reviewing prior neuroimaging studies, when available, remains crucial for accurate diagnosis in patients presenting with stroke.

## Conclusions

We reported a rare case of CVST associated with raloxifene therapy in a postmenopausal woman. Although raloxifene is widely used for osteoporosis and is known to increase the risk of venous thromboembolism, its association with CVST has not been previously documented. In evaluating patients with cerebrovascular events, clinicians should carefully consider medication histories along with laboratory findings and imaging studies. Clinicians should pay attention to CVST in postmenopausal patients presenting with atypical stroke symptoms while receiving raloxifene, especially in those with additional thrombotic risk factors such as immobility.
